# Daily oscillations in expression and responsiveness of Toll-like receptors in splenic immune cells

**DOI:** 10.1016/j.heliyon.2018.e00579

**Published:** 2018-03-20

**Authors:** Adam C. Silver, Sara M. Buckley, Michael E. Hughes, Andrew K. Hastings, Michael N. Nitabach, Erol Fikrig

**Affiliations:** a Department of Biology, University of Hartford, West Hartford, CT, USA; b Section of Infectious Diseases, Department of Internal Medicine, Yale University School of Medicine, New Haven, CT, USA; c Department of Cellular and Molecular Physiology, Yale University School of Medicine, New Haven, CT, USA; d Howard Hughes Medical Institute, Chevy Chase, MD, USA

**Keywords:** Immunology

## Abstract

Circadian rhythms refer to biologic processes that oscillate with an approximate 24-h period. These rhythms direct nearly all aspects of animal behavior and physiology. The aim of our study was to determine if Toll-like receptor (TLR) expression and responsiveness exhibit time-of-day dependent differences. Therefore, we isolated an adherent splenocyte population, which consisted primarily of B cells, dendritic cells, and macrophages, over the course of a 24-h light-dark period and measured daily changes in *Tlr1-8* mRNA levels and cytokine expression after cells were challenged at Zeitgeber time (ZT) 1 or ZT13 with a TLR ligand. In addition, we assessed TLR3 protein levels in adherent splenocytes over the 24-h light-dark period and challenged mice at ZT1 or ZT13 with poly(I:C), the TLR3 ligand. Our study revealed that in this adherent cell population, all *Tlrs* exhibited rhythmic expression except *Tlr2* and *Tlr5*, and all TLRs, except TLR8, demonstrated daily variations in responsiveness after challenge with their respective ligand. We also revealed that TLR3 protein levels fluctuate over the daily light-dark cycle in adherent splenocytes and mice exhibit a time-of-day dependent immune response when challenged with poly(I:C). Finally, we demonstrated that mRNA levels of *Tlr2* and *Tlr6* display rhythmic expression in splenic macrophages. Taken together, these findings could have important implications for TLR-directed therapeutics.

## 1 Introduction

Circadian (daily) rhythms refer to biological processes, from gene expression to behavior, that oscillate with an approximate 24-h period [1]. While external cues such as activity, food consumption, and temperature can direct daily rhythms, daily changes in light intensity entrain the suprachiasmatic nucleus (SCN, *i.e*., the master clock), more than any other environmental factor [2]. In mammals, the SCN, which is located in the hypothalamus, directs the oscillatory nature of many physiological and behavioral processes through the production of a multitude of neural and hormonal signals, which also synchronize peripheral circadian oscillators at the molecular level throughout the body [3, 4, 5]. On a molecular level, circadian rhythms are generated by a series of transcription-translation feedback loops [6].

Over the past decade it has become increasingly evident that the circadian clock has a vital hand in regulating various aspects, including both innate and adaptive parameters, of the immune system [7, 8]. We have previously demonstrated that the circadian clock directly modulates Toll-like receptor (TLR) 9 expression and responsiveness, which has time-of-day dependent implications regarding TLR9-mediated innate and adaptive immunity [9]. TLRs are a class of pattern recognition receptor (PRR) located in endosomal compartments or on the surface of certain immune cells that identify pathogen-associated molecular patterns (PAMPs), conserved microbial components that are exclusive to microorganisms. To date, 10 human TLRs (TLR1-10) and 12 mouse TLRs (TLR1-9 and TLR11-13) have been identified [10]. Each TLR recognizes and binds to a specific microbial ligand, which induces the production of proinflammatory cytokines and antimicrobial responses. In addition to initiating the innate immune response, TLR-PAMP recognition also directs the adaptive immune response [11].

The aim of this study was to determine whether other TLRs demonstrate daily fluctuations in expression and responsiveness. Therefore, we measured the expression of *Tlrs* (*Tlr1-8*) in an adherent splenocyte population, which predominately consisted of B cells, dendritic cells (DCs), and macrophages, as well as splenic macrophages isolated via magnetic cell sorting, over the daily light-dark (LD) cycle, subjected the former to PAMP challenges 12-h apart, and subsequently assessed cytokine mRNA levels. We then examined the daily fluctuations of TLR3 protein levels over the course of the LD cycle and challenged mice with the TLR3 ligand 12-h apart and subsequently assessed cytokine mRNA levels.

## 2 Materials and methods

### 2.1 Animals

Eight-week-old C57BL/6J male mice (The Jackson Laboratory) were fed rodent chow ad libitum, maintained under constant environmental conditions, and entrained to a 12-h light/12-h dark cycle (light period from 7:00 a.m. to 7:00 p.m.) for 2 weeks prior to experiments. Animals were euthanized at 8:00 a.m., 10:00 a.m., 2:00 p.m., 6:00 p.m., 8:00 p.m., 10:00 p.m., 2:00 a.m., and 6:00 a.m. These times correspond with Zeitgeber times (ZT) 1, 3, 7, 11, 13, 15, 19, and 23, respectively. Tissues were immediately collected for further processing as described below. During the study, animal care and treatment complied with National Institutes of Heath policy, were in accordance with institutional guidelines, and were approved by the University of Hartford or Yale University Animal Institutional Animal Care and Use committee.

### 2.2 Adherent splenocyte enrichment

Spleens were collected in RPMI 1640 (ThermoFisher) supplemented with 10% FBS. Each spleen was homogenized in 2 ml of medium, filtered through a 40 μM nylon cell strainer (BD Biosciences), added to a 75 cm^2^ cell culture flask (BD Biosciences) with 30 ml warm medium, and incubated at 37 °C in 5% CO_2_ for 1 h. Medium containing unattached cells was then removed and attached cells were washed two times with warm PBS. During the second wash step, the PBS was pipetted directly onto the cells five times. The adherent cells were scraped from the cell culture flask and resuspended in PBS [12].

### 2.3 Flow cytometry analysis of adherent splenocytes

Cells were fixed with 2% PFA for 10 min, spun down, resuspended in PBS and stored at 4 °C until analysis. Cells were washed two times with FACS buffer (PBS containing 2% FBS). Labeled with anti-CD3 (APC-Cy7 – Biolegend; Clone 17A2), anti-CD11b (Pacific Blue – Biolegend; Clone M1/70), anti-CD11c (Pe-Cy7 – BD Pharmingen; Clone HL3), and anti-CD19 (PE – Biolegend; Clone 6D5) antibodies for 1 h at room temperature, and washed twice with FACS buffer. They were then permeabilized and probed with TLR3 (APC - Biolegend; Clone 11F8) or a Rat IgG2a isotype (APC – Biolgend; Clone RTK2758) for 30 min at room temperature, and washed twice with FACS buffer. Samples were run on a BD LSRII flow cytometer and analyzed using FlowJo software, gating with the assistance of unstained and isotype stained samples.

### 2.4 *Ex vivo* TLR agonist challenge

Approximately 5 × 10^5^ cells per well were added to 12-well culture plates (Corning) and were challenged with 1 ml of medium containing Pam3CSK (500 ng/ml), heat-killed *Listeria monocytogenes* (HKLM, 5 × 10^7^), poly(I:C) (250 ng/ml), lipopolysaccharide (LPS, from *Escherichia coli* K12, 5 μg/ml), flagellin (FLA, from *Salmonella typhimurium*, 500 ng/ml), FSL-1 (500 ng/ml), ssRNA40 (1.25 μg/ml), imiquimod (R837) (5 μg/ml), or TL8-506 (100 ng/ml). TLR agonists were purchased from InvivoGen. After the plates were incubated at 37 °C in 5% CO_2_ for 3.5 h, the supernatant was removed, and the cells were washed with PBS. 300 μl of RLT buffer (Invitrogen) containing β-Mercaptoethanol was then added to each of the wells. Cell lysates were stored at −80 °C until RNA extraction was performed as described below.

### 2.5 *In vivo* poly(I:C) challenge

Mice were injected intraperitoneally with 30 μg poly(I:C) (InvivoGen) at either ZT1 or ZT13. 3 h after challenge, mice were sacrificed, spleens collected, and adherent splenocyte population isolated as described above. 600 μl of RLT buffer (Invitrogen) containing β-Mercaptoethanol was then added to the cell culture flask directly and cell lysates were stored at −80 °C until RNA extraction was performed as described below.

### 2.6 RNA extraction and quantitative PCR

RNA from adherent splenocytes was isolated using the RNeasy Mini kit (Qiagen) in conjunction with the RNase-Free DNase Set (Qiagen). cDNA was synthesized using the high capacity cDNA reverse transcription kit according to manufacturer's instructions (ThermoFisher). Relative quantitation of mRNA levels was performed by quantitative PCR via TaqMan Gene Expression Assays (ThermoFisher) and TaqMan Gene Expression Master Mix (ThermoFisher) using a StepOnePlus and a 7500 Fast System (ThermoFisher). Analyses were performed using the standard curve method with β-actin as the normalizing endogenous control. The following TaqMan Gene Expression Assays were used (ThermoFisher): *Tlr1* Mm00446095_m1, *Tlr2* Mm00442346_m1, *Tlr3* Mm01207404_m1, *Tlr4* Mm00445274_m1, *Tlr5* Mm00546288_s1, *Tlr6* Mm02529782_s1, *Tlr7* Mm00446590_m1, *Tlr8* Mm04209873_m1, *Per2* Mm00478113_m1, *Nr1d1* (*Rev-erbα*) Mm00520708_m1, *Actb* Mm00607939_s1, *Tnf* Mm00443258_m1, *Il6* Mm00446190_m1, *Ifnb* Mm00439522_s1, *Il1b* Mm00434228_m1.

### 2.7 TLR3 protein quantitation

Approximately half of the adherent splenocytes isolated from a single spleen, as described above, were spun down in a 1.5 ml microcentrifuge tube. After the supernatant was removed, cells were resuspended in 200 μl of T-PER Tissue Protein Extraction Reagent (Thermo Scientific), which included a protease inhibitor (Thermo Scientific). The sample was centrifuged at 10,000 × *g* for 5 min to pellet cell debris. The supernatant was collected and stored at −80 °C until analysis. TLR3 protein concentrations were determined via ELISA (Uscn Life Science Inc.), which were normalized to total protein concentration determined via BCA protein assay (Thermo Scientific).

### 2.8 Splenic macrophage isolation

Spleens were collected in RPMI 1640 (Invitrogen Life Technologies) supplemented with 10% FBS, homogenized in 1 ml of RMPI, filtered through a 70 μM nylon cell strainer (BD Biosciences) and spun down for 5 min. The supernatant was removed and cells were resuspended in 3 ml red cell lysing buffer (Sigma) and incubated for 10 min at room temperature. 7 ml of PBS were added to the cell suspension and cells were spun down for 5 min. The supernatant was removed, cells were resuspended in 3 ml PBS. A 1 ml aliquot was labeled with CD11b mouse microbeads (Miltenyi Biotech) for cell separation. The labeling was followed according to manufacturer's instructions and the autoMACS™ Pro Separator (Miltenyi Biotec) was utilized for the magnetic cell separation. The purity of the enriched fractions was assessed by flow cytometry using FITC-conjugated CD11b antibodies (Miltenyi Biotech). The enrichment method consistently yielded a purity of approximately 90% CD11b positive cells. Immediately after separation, enriched cells were lysed in 600 μl of RLT buffer (Invitrogen) containing β-Mercaptoethanol for later RNA extraction.

### 2.9 RNA extraction and quantitative PCR from enriched splenic macrophages

RNA from enriched macrophages was isolated using the RNeasy Plus-Micro kit (Qiagen). cDNA was synthesized using the high capacity cDNA reverse transcription kit (ThermoFisher). Due to low expression levels and a limited amount of cDNA, the TaqMan PreAmp Master Mix Kit (ThermoFisher) was used in conjunction with TaqMan Gene Expression Assays (ThermoFisher). The procedure was followed according to manufacturer's instructions. Relative quantitation of mRNA levels was performed as described above.

### 2.10 Statistical analysis

To statistically assess the rhythmicity of the data, we performed JTK_CYCLE analysis [13] implemented in R (x64 v2.12.1). Median-normalized qPCR expression levels of each transcript were tested for rhythmicity with a period length equal to 24 h. The R script used to run JTK_Cycle explicitly accounted for irregular sample spacing and biological replicates. Input data for TLR3 protein was formatted as six time points collected every 4 h over one complete day. For mRNA expression profiles, n varied between 7 and 9; for TLR3 protein profile, n was equal to 5. For the splenic macrophage isolated via CD11b magnetic microbeads, maximum-normalized qPCR expression levels were used and n varied between 8 and 15. A JTK_Cycle p-value adjusted for multiple testing of less than 0.01 was considered statistically significant. All R scripts are available on demand. One-way ANOVA with the Dunnett's posttest was used to assess differences between the acrophase and other time points. Data from the *ex vivo* and *in vivo* challenge experiments were analyzed using an unpaired two-tailed t test to assess differences between means at different time points via Prism 7.0a (GraphPad).

## 3 Results

### 3.1 *Tlr* expression levels over the daily light-dark cycle

Since we previously demonstrated that TLR9 is a direct target of the circadian molecular clock, we wanted to determine if the molecular clock also regulates other TLRs. We chose to examine TLR1-8 due to their relevance in human health (as humans do not possess TLRs 11, 12, and 13). Initially, we assessed if *Tlr* expression oscillates in a rhythmic fashion over the daily LD cycle in an adherent splenic cell population.

**Fig. 1:**
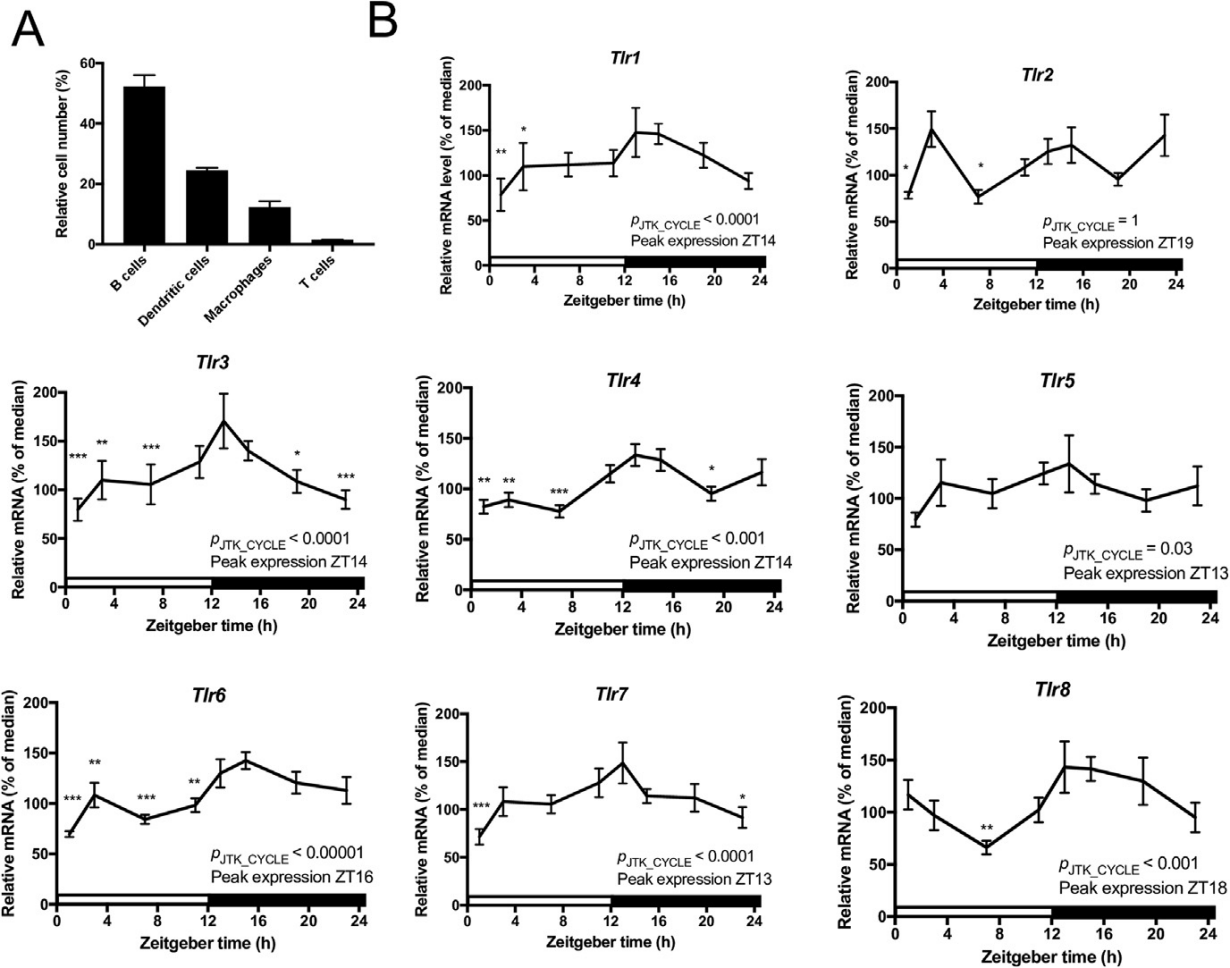
Daily variations of mRNA levels in adherent splenocytes. (A) Cellular composition in the adherent cell population. Cells were analyzed via FACS using CD19, CD11c, CD11b, and CD3 as characteristic surface markers of B cells, dendritic cells, macrophages, and T cells, respectively. For FACS analyses, n = 3. (B) Daily oscillations in *Tlr* gene expression in adherent cells from mouse spleen. Relative mRNA levels at each time point were determined by qPCR and calculated as the percentage of the median value over the 24-h period. Data are mean ± SEM of 7–9 animals per time point, compiled from 2 independent experiments, which produced similar results. One-way analysis of variance (ANOVA) was used to make comparisons between the acrophase and other time points. *p < 0.05, **p < 0.01, ***p < 0.001. JTK_CYCLE was used to statistically assess the rhythmicity of the data and calculate the time of peak expression. Open bar indicates light period, while colored bar indicates dark period.

Therefore, mice were entrained to the LD cycle for two weeks and quantitative PCR was used to assess mRNA levels from adherent cells obtained via plastic adherence over a 24-h period. The adherent cell population consisted predominately of macrophages (∼12%), DCs (∼25%), and B cells (∼53%) (Fig. 1A). The expression profiles for *Tlr1* and *Tlr6* were very similar, as mRNA levels were consistently low throughout the day and began to increase at the start of the active period, peaking at ZT14 (*p*_JTK_CYCLE_ < 0.0001; *p*_ANOVA_ < 0.01; Fig. 1B) and ZT16 (*p*_JTK_CYCLE_ < 0.00001; *p*_ANOVA_ < 0.0001; Fig. 1B), respectively. While *Tlr2* (*p*_JTK_CYCLE_ = 1; *p*_ANOVA_ < 0.01) and *Tlr5* (*p*_JTK_CYCLE_ = 0.03; *p*_ANOVA_ = 0.52) mRNA levels did not experience significant daily oscillations (Fig. 1B), we did observe a significant peak in *Tlr2* expression late in the mouse active phase (Fig. 1B). The expression profiles for *Tlr3* and *Tlr7* were nearly identical, as mRNA levels were low from ZT1 to ZT7, then increased significantly from ZT7 to ZT13, peaking at ZT14 (*p*_JTK_CYCLE_ < 0.0001; *p*_ANOVA_ < 0.001; Fig. 1B) and ZT13 (*p*_JTK_CYCLE_ < 0.0001; *p*_ANOVA_ < 0.01; Fig. 1B), respectively, and then gradually declining throughout the night. *Tlr4* expression levels were lowest from ZT1 to ZT7 (Fig. 1B) and gradually increased at the end of the day, peaking at ZT14 (*p*_JTK_CYCLE_ < 0.0001; *p*_ANOVA_ < 0.001; Fig. 1B). *Tlr8* mRNA levels were lowest at ZT7 and gradually increased through the end of the day and into the night, peaking at ZT18 (*p*_JTK_CYCLE_ < 0.001; *p*_ANOVA_ < 0.01; Fig. 1B).

### 3.2 *Ex vivo* daily variations in responsiveness to PAMPs

**Fig. 2:**
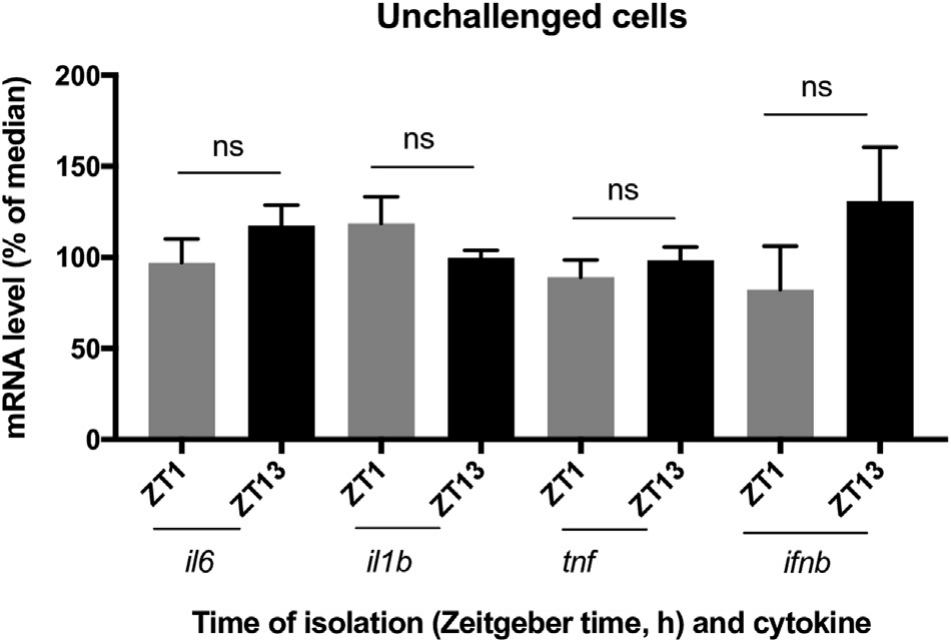
Cytokine levels in unchallenged splenic macrophages. Adherent splenocytes were isolated at ZT1 and ZT13 and relative cytokine mRNA levels (normalized to β-actin) were determined 3.5 h later by qPCR. Data are mean ± SEM of 9 animals per time point, compiled from 2 independent experiments and plotted as the percentage of the median value. *p < 0.05, significantly different as per two-tailed t test. ns, not significant.

We next evaluated whether the daily oscillations in *Tlr* mRNA levels would translate to daily variations in responsiveness to PAMPs. Therefore, adherent splenocytes were isolated at ZT1 and ZT13 and subsequently challenged with different PAMPs targeting specific TLRs. Cytokine (*il6*, *il1b*, *tnf*, and *ifnb*) mRNA levels were then assessed via quantitative PCR 3.5 h after challenge. In order to verify that differences in cytokine mRNA levels were due to time-of-day dependent differences in TLR responsiveness and not simply due to daily variations in basal cytokine expression, we assessed cytokine mRNA levels at ZT1 and ZT13 in unchallenged cells (Fig. 2). We did not observe statistically significant differences when comparing cytokine mRNA levels at ZT1 and ZT13 (Fig. 2).

**Fig. 3:**
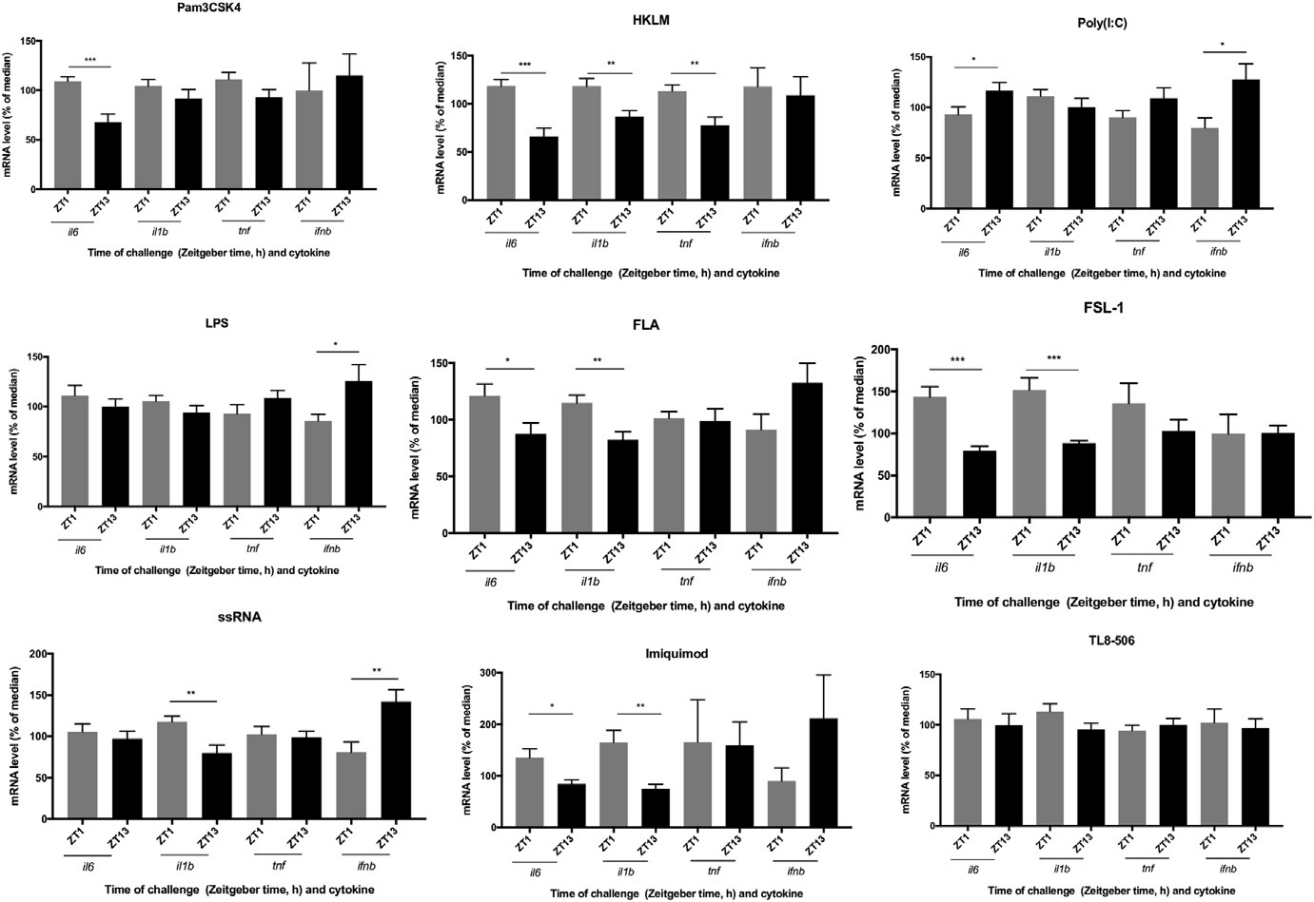
Daily variations in cytokine responses to TLR agonists in adherent splenocytes *ex vivo*. Adherent splenocytes were isolated at ZT1 and ZT13 and challenged with PAMPs targeting different TLRs. Relative cytokine mRNA levels (normalized to β-actin) were determined by qPCR 3.5 h after challenge. Data are mean ± SEM of 9 animals per time point, compiled from 2 independent experiments, which produced similar results, and plotted as the percentage of the median value (except n = 5 for imiquimod and TL8-506, which were from a third and fourth independent experiment). *p < 0.05, **p < 0.01, ***p < 0.001; significantly different as per two-tailed t test.

Since TLR2 forms a heterodimer with either TLR1 or TLR6, we challenged adherent splenocytes with a variety of PAMPs that are TLR1/2 (Pam3CSK4), TLR2 (heat-killed *Listeria monocytogenes*, HKLM), and TLR2/6 (FSL-1) specific. Adherent splenocytes challenged with the aforementioned PAMPs at ZT1 produced significantly higher cytokine mRNA amounts compared to ZT13 (Fig. 3), as Pam3CSK4 led to elevated *il6* levels (*p*_t test_ < 0.001; Fig. 3), HKLM produced increased *il6, il1b*, and *tnf* levels (*p*_t test_ < 0.001; *p*_t test_ < 0.01; *p*_t test_ < 0.01; Fig. 3), and FSL-1 generated higher *il6 and il1b* amounts (*p*_t test_ < 0.001; *p*_t test_ < 0.001; Fig. 3). After challenge with the TLR3 ligand, poly(I:C), we observed increased *il6* and *ifnb* mRNA levels at ZT13 compared to ZT1 (*p*_t test_ < 0.05; *p*_t test_ < 0.05; Fig. 3). Challenge with LPS, the TLR4 ligand, produced higher *ifnb* mRNA levels at ZT13 compared to ZT1 (*p*_t test_ < 0.05; Fig. 3). We observed higher *il6* and *il1b* mRNA levels at ZT1 compared to ZT13 after challenge with the TLR5 ligand, FLA (*p*_t test_ < 0.05; *p*_t test_ < 0.01; Fig. 3). We also challenged adherent splenocytes with two different TLR7 agonists, ssRNA40 and Imiquimod, the former activating TLR8 in humans [14]. ssRNA40 produced higher *il1b* mRNA levels at ZT1 (*p*_t test_ < 0.01; Fig. 3) but higher *ifnb* mRNA levels at ZT13 (*p*_t test_ < 0.01; Fig. 3), while we observed increased *il6* and *il1b* mRNA levels at ZT1 after challenge with Imiquimod (*p*_t test_ < 0.01; *p*_t test_ < 0.01; Fig. 3). We did not observe significant differences in cytokine expression between ZT1 and ZT13, when adherent splenocytes were challenged with TL8-506, the TLR8 agonist (Fig. 3).

### 3.3 *In vivo* daily variations in TLR3 protein levels and function

**Fig. 4:**
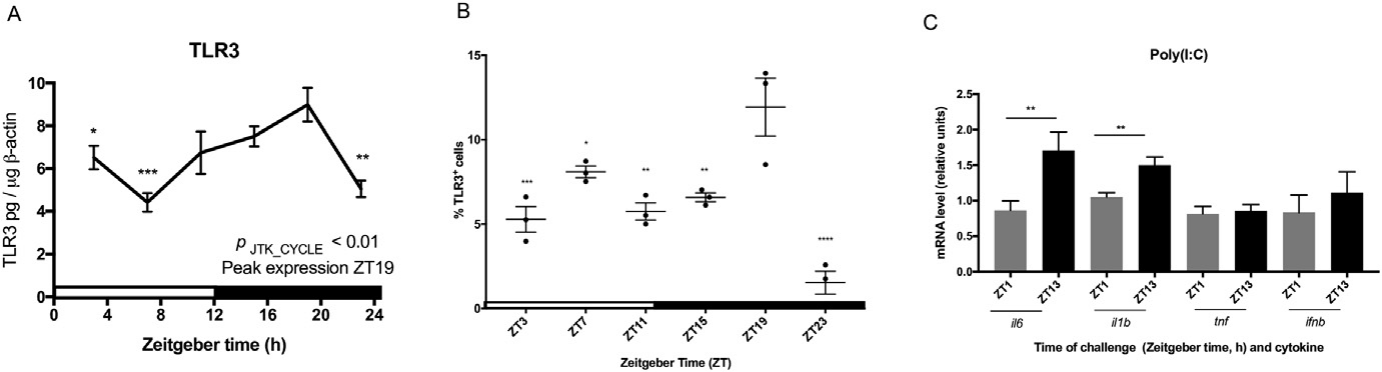
Daily variations in TLR3 protein amounts and responsiveness to poly(I:C) challenge *in vivo.* (A) Daily oscillation of TLR3 protein amounts in adherent splenocytes from mouse spleen collected every 4 h. TLR3 protein amounts at each time point were determined by ELISA and normalized to β-actin. Data are mean ± SEM of 5 animals per time point. One-way analysis of variance (ANOVA) was used to make comparisons between the acrophase and other time points. *p < 0.05, **p < 0.01, ***p < 0.001. JTK_CYCLE was used to statistically assess the rhythmicity of the data and calculate the time of peak expression. Open bar indicates light period, while colored bar indicates dark period. (B) Daily variation of percentage of TLR3-positive cells in an adherent splenocyte population collected every 4 h. TLR3-positive cells at each time point were determined by FACS. Data are mean ± SEM of 3 animals per time point. One-way analysis of variance (ANOVA) was used to make comparisons between the acrophase and other time points. *p < 0.05, **p < 0.01, ***p < 0.001, ****p < 0.0001. (C) Increased TLR3-mediated cytokine response at ZT13. Cytokine mRNA amounts were determined 3 h after poly(I:C) challenge in adherent splenocytes. Data are mean ± SEM of 10 animals per time point. *p < 0.05, **p < 0.01, ***p < 0.001; significantly different as per two-tailed t test.

Arboviruses (*e.g.,* West Nile virus and Dengue virus) are transmitted via vectors that display daily changes in biting activity [15, 16, 17]. It is plausible that innate immune recognition mechanisms have evolved to be at their peak responsiveness at the time of day when they are most likely to encounter a pathogen. Conversely, these viruses may take advantage of the vector's activity rhythm in order to infect the host at a time of day when it is most susceptible. Since TLR3 has previously been shown to play a role in arboviral detection [18, 19, 20], we specifically wanted to examine the daily variations of TLR3 in more detail. Therefore, we evaluated the existence of daily fluctuations of TLR3 protein levels in adherent splenocytes. TLR3 protein levels were lowest at ZT7 and then gradually increased over the subsequent 12 hours, peaking at ZT19 (*p*_JTK_CYCLE_ < 0.01; *p*_ANOVA_ < 0.001; Fig. 4A). In a subsequent experiment, we observed the highest percentage of TLR3-positive cells at ZT19 (*p*_ANOVA_ < 0.0001; Fig. 4B), which corresponds to the time of day when TLR3 protein levels were the highest (Fig. 4A). Finally, we examined whether the daily variations seen at the mRNA and protein level, as well as those seen during an *ex vivo* challenge, translated to time-dependent differences in responsiveness after an *in vivo* challenge. Therefore, we challenged mice at ZT1 or ZT13 and examined cytokine gene expression in adherent splenocytes. When mice were challenged with poly(I:C) at ZT13, they presented with increased expression of *il6* and *il1b* versus mice that were challenged at ZT1 (*p*_t test_ < 0.01; Fig. 4C).

### 3.4 *Tlr* expression levels over the daily light-dark cycle in splenic macrophages

**Fig. 5:**
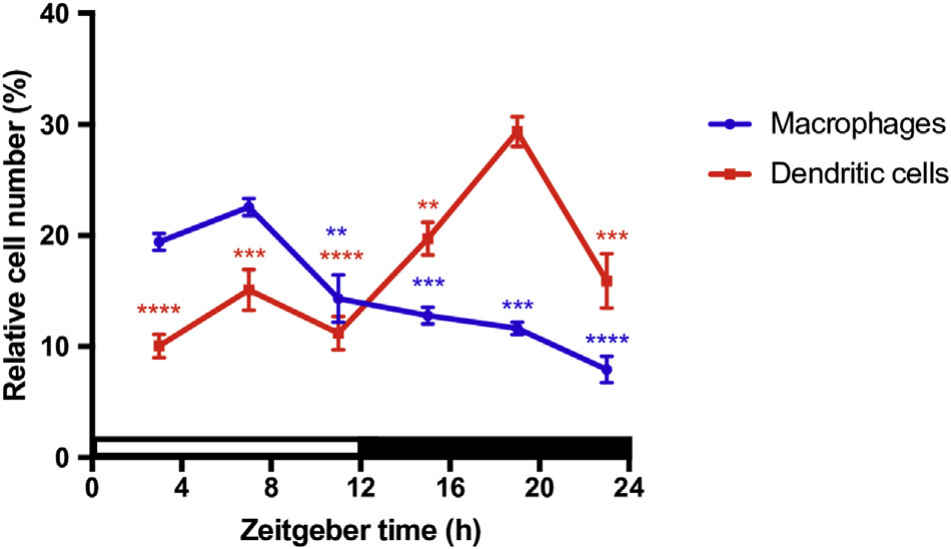
Daily variations in immune cells and *Tlr* mRNA levels in splenic macrophages. (A) Daily variation of the percentage of macrophages and DCs in an adherent splenocyte population. Cells were analyzed via FACS using CD11b and CD11c as characteristic surface markers of macrophages and DCs, respectively. Data are mean ± SEM of 3 animals per time point collected every 4 h. One-way analysis of variance (ANOVA) was used to make comparisons between the acrophase and other time points. *p < 0.05, **p < 0.01, ***p < 0.001, ****p < 0.0001.

**Fig. 6:**
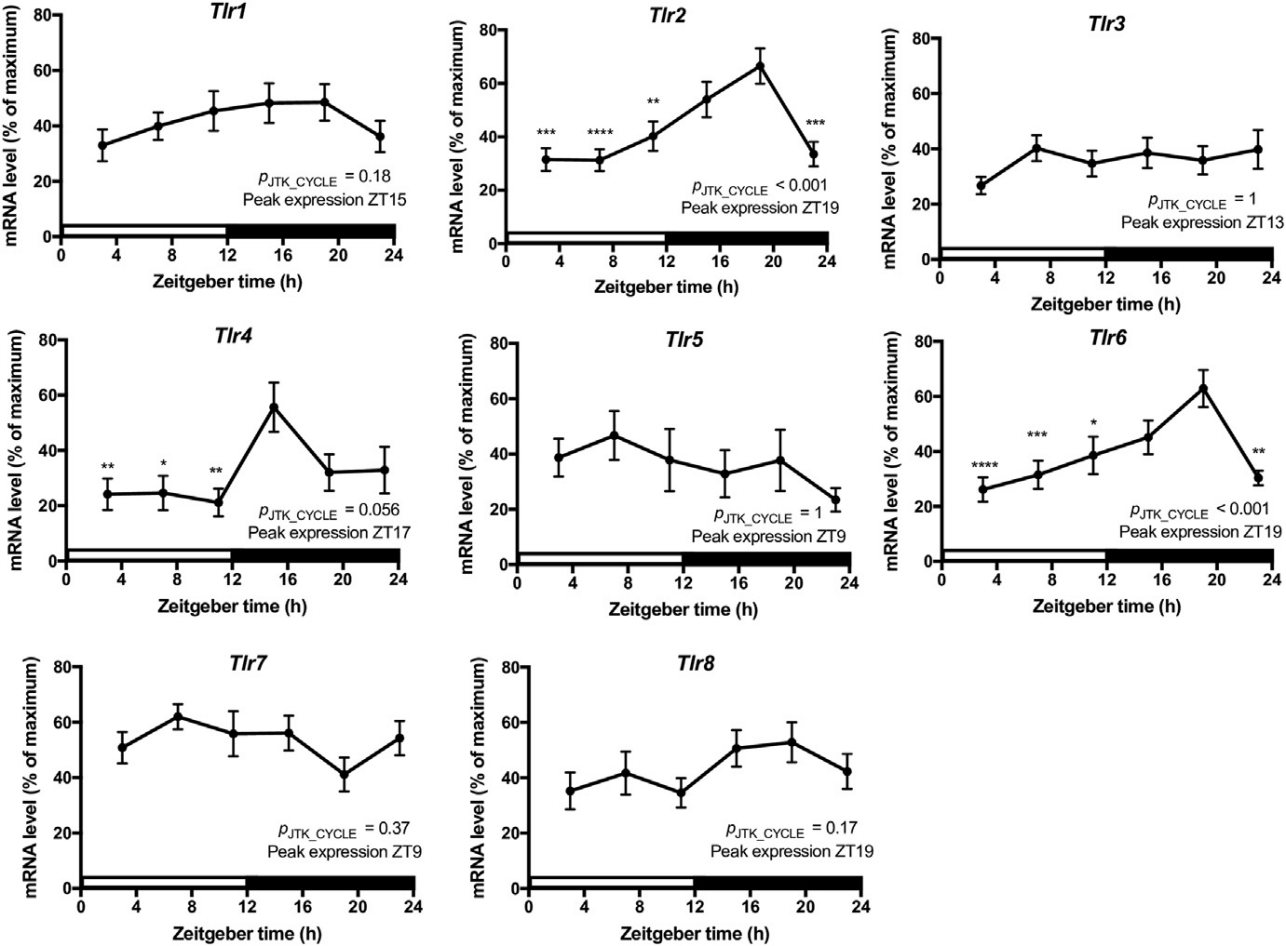
Daily oscillations in *Tlr* gene expression in splenic macrophages. Relative mRNA levels at each time point were determined by qPCR and calculated as the percentage of the maximum value over the 24-h period. Data are mean ± SEM of 12–15 animals per time point, compiled from 3 independent experiments. With the exception of *Tlr5*, which consisted of 8–10 animals per time point and was compiled from 2 independent experiments. One-way analysis of variance (ANOVA) was used to make comparisons between the acrophase and other time points. *p < 0.05, **p < 0.01, ***p < 0.001. JTK_CYCLE was used to statistically assess the rhythmicity of the data and calculate the time of peak expression. Open bar indicates light period, while colored bar indicates dark period.

Splenic immune cell trafficking is regulated in a circadian fashion, as the total number of splenocytes displays circadian rhythmicity, with the relative number of T cells and macrophages and the absolute number of B cells and macrophages fluctuating over the course of the daily cycle [21]. Given that daily variations in cell trafficking could account for the observed changes in gene expression, we assessed the percentage of macrophages and DCs in the adherent splenic cell population over the 24-h period. The percentage of macrophages peaked at ZT7 and steadily declined throughout the mouse active phase (*p*_ANOVA_ < 0.0001; Fig. 5), while conversely, DCs were highest during the mouse active phase, peaking at ZT19 (*p*_ANOVA_ < 0.0001; Fig. 5). In order to tease apart the *Tlr* rhythms observed within the adherent splenic cell population, we isolated splenic macrophages using CD11b magnetic microbeads every 4 h over the course of the 24-h LD period and examined mRNA levels using qPCR. The expression profiles for *Tlr2* and *Tlr6* were very similar, as mRNA levels were consistently low throughout the day and began to increase at the start of the active period, peaking at ZT19 (*p*_JTK_CYCLE_ < 0.001; *p*_ANOVA_ < 0.0001 and *p*_ANOVA_ < 0.001, respectively) (Fig. 6). While mRNA levels for the other *Tlrs* did not exhibit rhythmic oscillations, *Tlr4* expression displayed a significant expression peak at ZT15 (*p*_JTK_CYCLE_ = 0.056; *p*_ANOVA_ < 0.01) (Fig. 6).

## 4 Discussion

Several aspects of the immune system operate in a time-dependent manner [7, 8]. Elucidating additional immune mechanisms and determining their time of peak responsiveness could have great consequences in disease treatment (*i.e.,* various inflammatory diseases) and vaccine efficacy. While we previously revealed that TLR9 is a direct target of the circadian molecular clock [9], here, we demonstrate that additional TLRs could also be regulated in a time-of-day dependent manner.

**Fig. 7:**
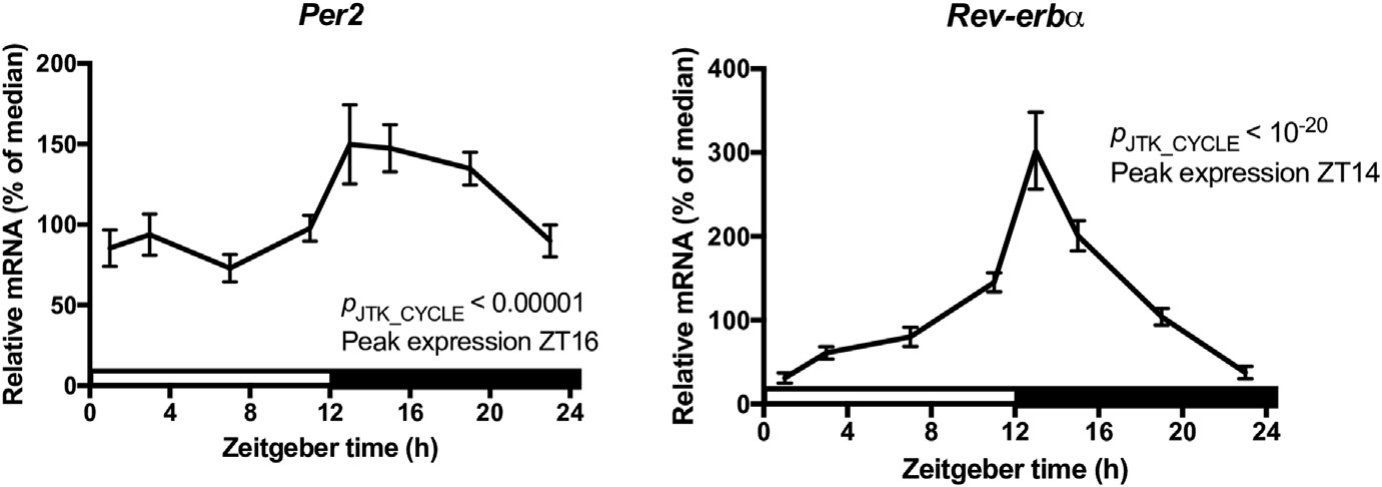
Daily variations of mRNA levels in adherent splenocytes. Daily oscillations in clock gene expression in adherent splenocytes isolated from mouse spleen. Relative mRNA levels at each time point were determined by qPCR and calculated as the percentage of the median value over the 24-h period. Data are mean ± SEM of 7–9 animals per time point, compiled from 2 independent experiments. Open bar indicates light period, while colored bar indicates dark period.

The major cell constituents of the mouse spleen are B cells (∼58%), T cells (∼21%), DCs (∼5%), and macrophages (∼4%) [22]. The splenocyte population we examined was enriched in both DCs (∼17) and macrophages (∼15), while B cells (∼53%) remained approximately the same and T cells were depleted (<2%) (Figs. 1A and 5). In order to verify our experimental animals were properly entrained, we assessed the rhythmicity of the clock genes *Per2* and *Rev-erbα* in this population of cells*.* We observed robust oscillations of mRNA for both genes (Fig. 7) and the expression profiles were consistent with what was previously observed in splenocytes [23] as well as enriched splenic macrophages, B cells, and DCs isolated via magnetic cell sorting [24]. We revealed that with the exception of *Tlr2* and *Tlr5*, all other *Tlrs* experienced daily rhythmicity in their mRNA levels (Fig. 1B). Expression of these six *Tlrs* was predicted to peak during the mouse active phase from ZT13 to ZT18. We also observed that all TLRs, except TLR8, demonstrated daily variations in responsiveness after challenge with their respective ligand. However, the time of increased responsiveness varied for the TLR agonist and the cytokine that was examined (Fig. 3). Despite *Tlr* expression levels predicted to peak during the mouse active phase, we observed increased responsiveness during the beginning of the mouse rest period (ZT1), for several TLRs and their respective ligands (Figs. 1B and 3). The discrepancy between expression and responsiveness could potentially be explained by several factors, such as a lag between mRNA and protein levels (*e.g.,* TLR3; Figs. 1B and 4), circadian regulation of post-translational modifications, TLR cellular trafficking, or that various aspects of the TLR signaling pathway [21] could be under circadian control. In addition, since we assessed responsiveness at only two timepoints, it is highly plausible that the peak and nadir were at a time other than ZT1 or ZT13.

TLR2 ligand recognition occurs after its heterodimerization with TLR1 or TLR6 [25]. The expression profiles for *Tlr1* and *Tlr6* were similar (Fig. 1B), while *Tlr2* levels did not display daily rhythmicity (Fig. 1B). Interestingly, despite the lack of daily cycling of *Tlr2* mRNA, we still observed daily variations in TLR2 responsiveness to Pam3CSK4, HKLM, and FSL-1, as cytokine levels were elevated at ZT1 compared to ZT13 (Fig. 1B and 3). This could potentially be due to *Tlr2* levels oscillating with a different period length, TLR2 protein levels could remain relatively constant throughout the day and the variation in responsiveness is mediated through the cycling of *Tlr1* and *Tlr6*, or perhaps *Tlr2* is regulated post transcriptionally in a circadian fashion.

TLR7 and TLR8 are genetically and functionally related, as they have a role in both bacterial and viral recognition [26]. The agonists, ssRNA40 and imiquimod, which are an RNA oligonucleotide and a synthetic chemical that possesses antiviral effects, respectively, were used to stimulate TLR7 [27,28]. Both agonists produced significantly higher *ilb1* expression when challenged at ZT1, yet ssRNA40 challenge produced significantly higher *ifnb* levels when challenged at ZT13, while imiquimod elicited higher *il6* levels at ZT1 (Fig. 3). Interestingly, we did not observe any time-of-day dependent differences in cytokine responses when adherent splenocytes were challenged with the TLR8 ligand, TL8-506 (Fig. 3). This does not eliminate the possibility that TLR8 responsiveness varies over the daily cycle, as it is highly plausible that the nadir and/or peak is a time other than ZT 1 or ZT13.

TLR3 is an endosomally located TLR that recognizes nucleic acids, specifically, dsRNA, which is associated with viral infection [29]. We revealed *Tlr3* mRNA and protein levels undergo significant daily oscillations in adherent splenocytes (Figs. 1B and 4A), which translated to daily variations in TLR3 responsiveness (Fig. 3). We then wanted to determine if these time-of-day dependent differences in TLR3 responsiveness *ex vivo* would be recapitulated *in vivo*. Therefore, when mice were challenged with poly(I:C) at ZT13, they presented with higher adherent splenocyte mRNA expression of *il6* and *il1b*, 3 h after challenge compared to mice that were challenged at ZT1 (Fig. 4B), which suggests that peak TLR3 responsiveness is dependent upon the time of day when the agonist is encountered.

Our findings reinforce numerous *in vivo* reports citing time-of-day dependent responses to various pathogen or PAMP challenges and it is established that these infection models trigger TLR activation. For example, mice challenged with *L. monocytogenes* (TLR2-dependent response) possessed increased chemokine and cytokine levels, and a lower bacterial load depending on the time of day when challenge occurred [30, 31]. LPS challenge (TLR4-dependent response) produced a daily susceptibility rhythm *in vivo* [32, 33] and immune cells produced daily fluctuations in cytokine response to *in vivo* and *ex vivo* LPS challenge [21, 34]. It was also shown that mice infected with *Salmonella* Typhimurium (TLR4 and TLR5-dendent responses) or challenged with a TLR5 agonist, experienced differences in bacterial burdens and inflammatory responses that were dependent upon the time of day when they were challenged, respectively [33, 35]. In the present study, we revealed that *Tlr5* mRNA did not experience daily oscillations, however, we did observe significant daily variations in *il6* and *il1b* expression after FLA challenge (Figs. 1B and 3).

Since splenic cellular composition changes throughout the day [21] and splenic macrophages, B cells, and DCs possess functional molecular clocks [24], we wanted to investigate macrophage contribution to the *Tlr* rhythms we observed in a heterogeneous population containing the aforementioned immune cells. In splenic macrophages, mRNA levels from only *Tlr2* and *Tlr6* displayed significant daily oscillations (Fig. 6), compared to *Tlr1, Tlr3, Tlr4, Tlr6, Tlr7,* and *Tlr8* in an adherent cell population (Fig. 1B). This further demonstrates the complexity and intricacies of the clock, as each cell type within the spleen can potentially be contributing a unique expression rhythm while its relative cell number could also be fluctuating in a daily fashion. For example, as previously mentioned, TLR2 and TLR6 form a heterodimer, therefore, it is interesting that their expression rhythms are almost indistinguishable from each other in splenic macrophages (Fig. 6). In addition, their rhythms are antiparallel to the rhythmic fluctuation of the relative and absolute number of splenic macrophages, which could represent an evolutionary mechanism to compensate for the decrease in splenic macrophage numbers during the mouse active phase [21] (Figs. 5 and 6).

Chronotherapeutics involves the consideration of the timing of treatment administration when determining efficacy. It was recently shown that a majority of the top 100 best-selling drugs in the United States target circadian genes [36] and therefore, the time of day when the drug is taken should be considered. Over the past decade, the extent to which the molecular clock influences the immune system has become evident as a multitude of immune parameters fluctuate over the 24-h period [7, 8, 37]. Consequently, this could give rise to chrono-immunotherapy as daily oscillations of immune response mechanisms can be taken advantage of in order to maximize vaccine or drug efficacy. For example, it was shown that influenza vaccine administered in the morning produced higher antibody responses compared to the afternoon [38]. We previously reported that TLR9 expression and function is controlled by the circadian molecular clock, furthermore, when mice were immunized with the TLR9 ligand at the time of peak responsiveness, they presented weeks later with a heightened adaptive immune response [9]. In our current study, our data suggest the molecular clock plays a role in modulating TLR time-of-day dependent responses. Taken together, as several TLR ligands are currently being evaluated for their effectiveness as vaccine adjuvants [39] in order to achieve maximum efficacy, time of day for immunization should potentially be considered. In addition to considering their role in vaccine development, TLRs also impact certain inflammatory diseases and as a consequence, TLR agonists are being explored in order to inhibit their activation and thereby prevent an enhanced inflammatory state [39, 40]. Once again, the time of day the agonist is administered, potentially during peak TLR responsiveness, should be considered in order to achieve greatest efficacy.

## Declarations

### Author contribution statement

Adam C. Silver: Conceived and designed the experiments; Performed the experiments; Analyzed and interpreted the data; Contributed reagents, materials, analysis tools or data; Wrote the paper.

Sara M. Buckley: Performed the experiments.

Michael E. Hughes: Analyzed and interpreted the data.

Andrew K. Hastings: Performed the experiments; Analyzed and interpreted the data.

Michael N. Nitabach, Erol Fikrig: Contributed reagents, materials, analysis tools or data.

### Funding statement

This work was supported by Faculty Research grants from the College of Arts and Sciences Dean's Office at the University of Hartford (to A.S.).

### Competing interest statement

The authors declare no conflict of interest.

### Additional information

No additional information is available for this paper.
